# No miRNA were found in *Plasmodium *and the ones identified in erythrocytes could not be correlated with infection

**DOI:** 10.1186/1475-2875-7-47

**Published:** 2008-03-10

**Authors:** Xiangyang Xue, Qingfeng Zhang, Yufu Huang, Le Feng, Weiqing Pan

**Affiliations:** 1 Institute for Infectious Diseases and Vaccine Development, Tongji University College of Medicine, 1239 Siping Road, Shanghai 200092, China; 2 Department of Pathogenic Biology, Second Military Medical University, 800 Xiang Yin Road, Shanghai 200433, China

## Abstract

**Background:**

The transcriptional regulation of *Plasmodium *during its complex life cycle requires sequential activation and/or repression of different genetic programmes. MicroRNAs (miRNAs) are a highly conserved class of non-coding RNAs that are important in regulating diverse cellular functions by sequence-specific inhibition of gene expression. What is know about double-stranded RNA-mediated gene silencing (RNAi) and posttranscriptional gene silencing (PTGS) in *Plasmodium *parasites entice us to speculate whether miRNAs can also function in *Plasmodium*-infected RBCs.

**Results:**

Of 132 small RNA sequences, no *Plasmodium*-specific miRNAs have been found. However, a human miRNA, miR-451, was highly expressed, comprising approximately one third of the total identified miRNAs. Further analysis of miR-451 expression and malaria infection showed no association between the accumulation of miR-451 in *Plasmodium falciparum*-iRBCs, the life cycle stage of *P. falciparum *in the erythrocyte, or of *P. berghei *in mice. Moreover, treatment with an antisense oligonucleotide to miR-451 had no significant effect on the growth of the erythrocytic-stage *P. falciparum*.

**Methods:**

Short RNAs from a mixed-stage of *P. falciparum*-iRBC were separated in a denaturing polyacrylamide gel and cloned into T vectors to create a cDNA library. Individual clones were then sequenced and further analysed by bioinformatics prediction to discover probable miRNAs in *P. falciparum*-iRBC. The association between miR-451 expression and the parasite were analysed by Northern blotting and antisense oligonucleotide (ASO) of miR-451.

**Conclusion:**

These results contribute to eliminate the probability of miRNAs in *P. falciparum*. The absence of miRNA in *P. falciparum *could be correlated with absence of argonaute/dicer genes. In addition, the miR-451 accumulation in *Plasmodium*-infected RBCs is independent of parasite infection. Its accumulation might be only the residual of erythroid differentiation or a component to maintain the normal function of mature RBCs.

## Background

MicroRNAs (miRNAs), a newly discovered class of endogenous ~21 nucleotide regulatory non-coding small RNAs, post-transcriptionally regulated gene expression in eukaryotes by targeted RNA degradation and translational arrest [1]. Similarly to short interfering RNAs (siRNAs), miRNAs are produced in the cytoplasm from a precursor, which contains an imperfectly matched inverted repeat forming a partial double-stranded region, by the ribonuclease, dicer. One strand of the resulting miRNA duplex intermediate is then recruited by the argonaute nuclease, an enzyme involved in the silencing mechanism, and incorporated into the RNA-induced silencing complex (RISC). The mature single-stranded miRNA guides the target RISC to bind to the 3' untranslated regions of target mRNAs based on sequence complementarity, which are then degraded and/or translationally inhibited. Since the discovery of the founding members of the miRNA family, *lin-4 *and *let-7*[2,3], hundreds of miRNAs had been identified in viruses, plants, and animals by molecular cloning and bioinformatic approaches [4-7].

The malaria parasite *Plasmodium falciparum *is a eukaryote with a complex life cycle in two hosts and a unique repertoire of proteins expressed at distinct life cycle stages. Its genome is strikingly A-T rich (80%–90%) [8,9]. The paucity of annotated transcription factors and the phased expression of blood stage transcripts have led to the proposal that post-transcriptional gene silencing is an important mechanism in the regulation of gene expression in *Plasmodium *[10]. Moreover, the phenomenon of double-stranded RNA-mediated gene silencing (RNAi) in the *Plasmodium *parasite had been reported in several studies [11-15]. In addition, ~21 nucleotide siRNAs were found in other protozoa such as *Tetrahymena thermophila*, *Trypanoma brucei*, and *Entamoeba histolytica*[16,17]. In *Entamoeba histolytica*, the presence of siRNAs was investigated, and seventeen putative miRNA candidates were identified by bioinformatics methods [18]. However, bioinformatics analysis of the *P. falciparum *genome showed that the genes encoding dicer and argonaute, which are crucial components in the RNAi pathway and play a key role in miRNA biogenesis and functional pathways, were absent [10,19,20].

In this study, a possibility of the miRNAs present in *P. falciparum *was investigated. Consistent with a recent report [21], *P. falciparum*-specific miRNAs were not found. However, Short RNAs from infected red blood cells (iRBCs), including miRNAs that matched human genomic sequences, particularly showed an abundant human miRNA in iRBC. The associations between the generation of these miRNAs and the infection of *Plasmodium *were explored.

## Methods

### Parasites, animals, and reagents

The 3D7 strain of *P. falciparum *was cultured *in vitro *using group O blood according to Trager's method[22]. A vial containing *P. berghei *ANKA strain frozen in liquid nitrogen was thawed quickly and injected intraperitoneally with 10^7 ^parasitized erythrocyte into KM mice with age of 6~8 weeks. When the parasitaemia in the mice reached approximately 10%, the blood was harvested for detecting expression of miR-451. Thin blood smears were prepared to determine parasitaemia.

### Small RNA preparation, cloning and analysis

The cloning and analysis of small RNAs was performed as described previously[5]. Briefly, total RNAs were isolated using Trizol reagent (Invitrogen) from a mixed-stage of the parasite including ring stage, trophazoite and schizoint when the parasitemia reached approximately 10%, and short RNAs 18 to 25 nucleotides in length were purified by 15% PAGE with 8 M urea. This approach is expected to clone all cellular miRNAs, including miRNAs from erythrocytes and *Plasmodium*, as well as any fragments of cellular mRNAs or noncoding RNAs that fall into this size range. After ligating 3' and 5' adapters (Takara, Dalian, China) to the small RNAs, the resultant miRNAs could be amplified by standard RT-PCR. The cDNAs then were concatenated, cloned and sequenced. The annotation was based on information from GenBank [23], a database of *P. falciparum genome *[24], *a dataset of human tRNA sequences*[25], *and a database of miRNAs*[26]. The genomic regions containing candidate sequences with ~70 bp flanking sequences were used to predict secondary structures using RNA-fold software such as Mfold[27].

### Northern blot analysis

Total RNAs were subjected to electrophoresis on a 15% polyacrylamide gel under denaturing conditions, and the separated molecules were transferred electrophoretically to a Hybond-N nylon membrane (Pall-Gelman, USA). The membranes were hybridized with a biotin-labeled oligonucleotide probe (Invitrogen, Shanghai, China) corresponding to mature miRNA sequences. After incubation with streptavidin-horseradish peroxidase conjugate and an enhanced light-based chemiluminescent substrate detection system (Pierce), signals were detected by covering the membrane with plastic wrap and exposing to X-ray film.

### Synthesis and administration of miR-451 antisense oligos (ASO)

The unconjugated locked nucleic acids (LNA)-modified oligonucleotides (Sangon, Shanghai, China) were synthesized as the substitution of every third nucleotide position by LNA. The anti-miR-451 LNA oligonucleotide 5'-aaActCagTaaTggTaaCggTtt-3' (uppercase: LNA; lowercase: DNA) was complementary to the mature miR-451 sequence. The substance was formulated in RPMI 1640 medium containing 25 mM HEPES, 24 mM NaHCO3, and 15% (vol/vol) heat-inactivated rabbit serum to final concentration of 10, 100, 500 and 1000 nM for *P. falciparum *cultivation. Highly-synchronized *P. falciparum*-infected erythrocytes were adjusted to the original culture with approximately 2% hematocrit and 0.5% parasitaemia. After incubating with different concentrations of the miR-451 ASO for 72 hr, thin blood smears were prepared to determine parasitaemia of each sample by counting the number of parasites in 2,000 erythrocytes.

## Results

### Cloning of short RNAs from *P. falciparum*-infected RBCs

**Figure 1:**
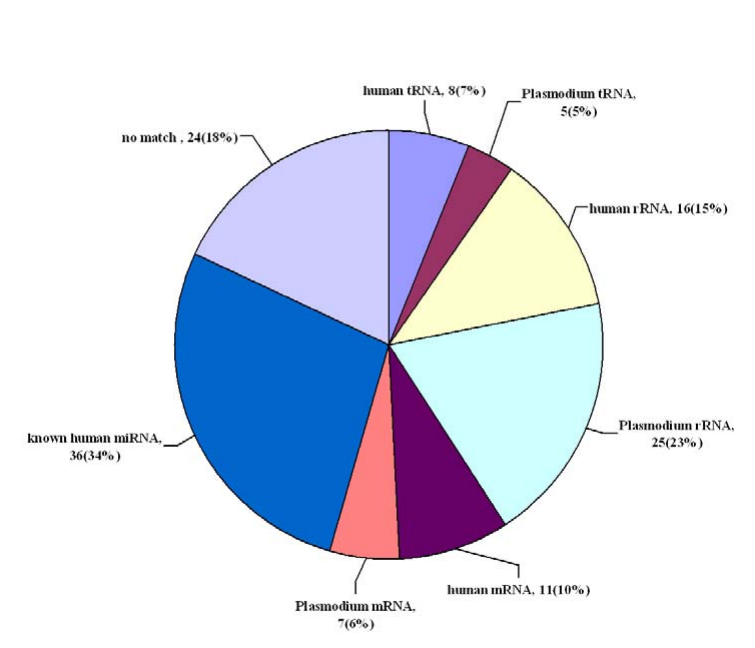
**Distribution of cloned short RNAs from *P. falciparum*-iRBC**. One-hundred thirty-two short RNAs 18 to 26 nucleotides in length were isolated from *P. falciparum*-iRBCs and grouped according to sequence annotation (see Materials and Methods for details). Those with more than three mismatches to human or *Plasmodium *sequences were classified as ???.

Total 132 short RNAs 18–26 nucleotides in length were obtained through cloning of cDNAs from *P. falciparum*-infected RBCs. Of the 132 short RNAs, 54 (40.91%) were rRNAs and tRNAs from human blood and *Plasmodium*, 18 (13.64%) were degraded fragments of human blood and *Plasmodium *mRNA, 36 (27.27%) were human miRNAs, and 24 (18.18%) did not match the human nor *Plasmodium *genome. Consistent with recent reports[21], no *Plasmodium*-specific miRNAs sequences were identified(Figure 1). The human miR-451 accumulated at a very high level, comprising almost 1/3 of the identified miRNAs isolated from *P. falciparum*-iRBCs (Table 1). Northern blot analysis confirmed the abundance of miR-451 in *Plasmodium*-infected RBCs.

**Table 1:** Frequencies of known miRNAs identified from *P. falciparum*-iRBCs

**miRNAs**	**Number of clones**	**Percentage(%)**
**hsa-let-7a**	1	2.78
**hsa-let-7b**	7	19.44
**hsa-let-7f**	1	2.78
**hsa-mir-16**	6	16.67
**hsa-mir-91**	2	5.56
**hsa-mir-92**	1	2.78
**hsa-mir-106**	1	2.78
**hsa-mir-142**	2	5.56
**hsa-mir-144**	2	5.56
**hsa-mir-451**	14	38.89
**Total**	36	100

### The association between miR-451 expression level and the development of erythrocyte-stage *P. falciparum*

**Figure 2:**
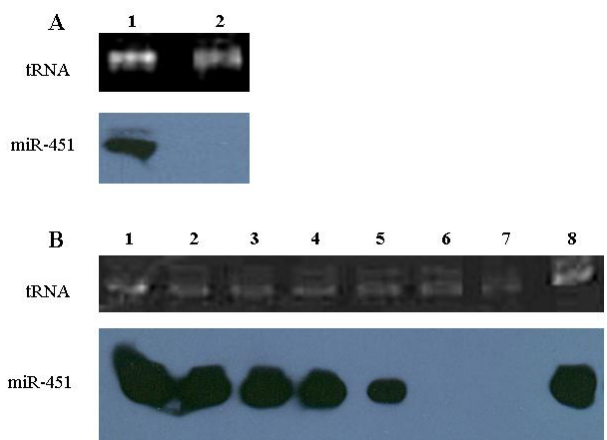
**Detection of expression of miR-451 by Northern blot**. (A) **Analysis of miR-451 expression in human WBCs and *P. falciprum*-iRBCs**. Total RNA (2 μg) from WBCs and *P. falciprum*-iRBCs was separated by denaturing PAGE and blotted to a nylon membrane. Biotin-labeled miR-451 was used as a hybridization probe. Lane 1, infected RBC; Lane 2, isolated WBCs. (B) **Analysis of miR-451 expression in different erythrocytic-stage *P. falciparum*-iRBCs**. Total RNA (2 μg) from known numbers of cells were prepared at 24 h intervals from a single tightly synchronized culture. Parasite stage was determined by thin blood smears. Lane 1, healthy human RBCs; lane 2, 0-h culture after tight synchronization (ring-stage iRBCs); lane 3, 24-h culture (late trophozoite-stage iRBCs); lane 4, 48-h culture (schizont-stage iRBCs); lane 5, 72-h culture (new reproductive cycle of *P. falciparum*); lane 6, purified *P. falciparum*; lane 7, purified *P. falciparum *treated with RNase H to remove residual RNA contamination; lane 8, 72-h culture of *P. falciparum*-iRBCs loaded with equivalent numbers of erythrocytes as lane 2.

The specificity of miR-451 expression was firstly investigated. To eliminate the possibility that the accumulated miR-451 in *P. falciparum*-iRBCs were derived from contamination by human white blood cells (WBCs), the transcription of miR-451 in parasite-iRBC and human WBCs was analysed by Northern blot. The result showed that miR-451 was transcribed at a very high level in *P. falciparum*-iRBCs, whereas no miR-451 transcripts were observed in human WBCs (Figure 2A).

The malaria parasite in infected erythrocytes has several developmental stages, including the early ring trophozoite, late trophozoite, and schizont. To investigate the association between the expression level of miR-451 and parasite development, the transcription of miR-451 at different development stages of synchronized *P. falciparum*-infected RBCs was assayed by Northern blot. Compared to equivalent amounts of total RNA (2 μg) per lane, there were no significant difference in accumulated miR-451 transcription levels from rings to pigmented trophozoite stages. Moreover, when total RNA from equivalent numbers of erythrocytes was loaded, there was also no significant difference among them, although the transcription level of miR-451 seemed slightly lower in the next generation (cultivated after 72 h) (Figure 2B).

To further analyse the potential functions of miR-451 in the development of erythrocyte-stage parasites, the *Plasmodium*-infected RBCs were synchronized and cultured in RPMI-1640 medium containing an ASO of miR-451 (Sangon, Shanghai, China)[22]. Different concentrations of the miR-451 ASO were added to the cultures and parasitaemias were determined. No interfering effect of the ASO of miR-451 on the parasite growth was observed compared to the untreated group.

### Expression of miR-451 in *P. berghei*-infected mouse erythrocytes

**Figure 3:**
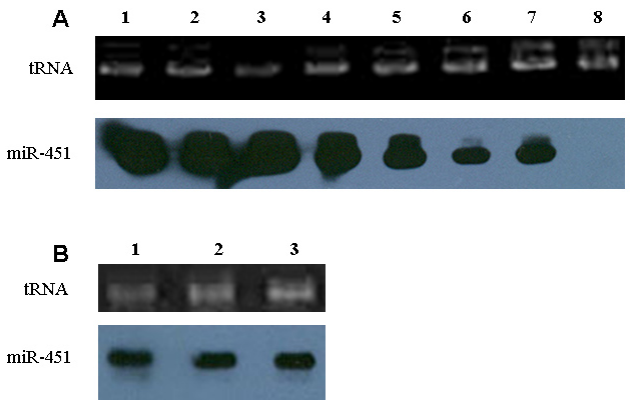
**Analysis of miR-451 expression in the blood of *P. berghei*-infected KM mice at different parasitaemias**. Female KM mice (6–8 weeks old) were infected with the blood stages of *P. berghei *(ANKA strain) by intraperitoneal injection of 10^5 ^infected erythrocytes. The course of infection was monitored by examination of Giemsa-stained blood films. (A) Total RNAs from the whole blood of uninfected and infected mice with different parasitaemias were analysed by Northern blotting. Lane 1, whole healthy blood; lane 2, 0.5% parasitaemia; lane 3, 2% parasitaemia; lane 4, 12% parasitaemia; lane 5, 28% parasitaemia; lane 6, 43% parasitaemia; lane 7, 60% parasitaemia; lane 8, 86% parasitaemia. (B) Total RNAs from equivalent numbers of blood cells (10^7^) with different parasitaemias were analysed by Northern blotting. Lane 1, whole healthy blood; lane 2, 43% parasitaemia; lane 3, 60% parasitaemia.

In addition to *Homo sapiens*, miR-451 is also expressed in other species, such as *Mus musculus, Rattus norvegicus, Danio rerio, Xenopus tropicalis, Gallus gallus, and Monodelphis domestica *[26]. In this study, the expression of miR-451 in the blood of KM mice was confirmed by Northern blot analysis (Figure 3). The investigation whether the expression of miR-451 could be influenced by *Plasmodium *infection *in vivo *was performed by using the *P. berghei *(ANKA strain) mice model. The results showed that miR-451 expression, when comparing equivalent amounts of RNA, decreased slightly from lower to higher parasitaemia and became undetectable at 86% parasitaemia (Figure 3A). However, if the total RNAs loaded were adjusted based on the number of blood cells, the difference in miR-451 transcript level disappeared (Figure 3B). This indicated that the weak signal of the miR-451 transcript in samples with higher parasitaemia was due to dilution of parasite RNAs.

## Discussion

Eukaryotes with a complex life cycle may be particularly likely to express miRNAs, as they could regulate their gene expression at different stages of their life cycle or affect host cell factors that increase the likelihood of maintaining long-term infection in the host. miRNAs from the host cell may also participate in the regulation of parasite gene expression even if *Plasmodium *cannot produce its own miRNAs. Therefore, miRNAs present in *Plasmodium*-infected RBCs were examined by using a short RNA cloning approach. Similar to the results of Rathjen *et al *[21], no any *Plasmodium*-specific miRNAs was detected, further eliminating the probability of miRNAs in *P. falciparum*. If any regulatory RNAs exist in *P. falciparum*, they will be distinct from miRNAs. However, some known human miRNAs did isolate. Notably, the miRNA miR-451 accumulated to a very high level in infected RBCs, comprising more than one third of the cloned miRNAs. So the relationship between malaria infection and the expression of miR-451 were further explored.

The data presented here showed that accumulation of miR-451 in RBCs is unrelated to the life cycle of *P. falciparum *in the erythrocytic stage and parasitaemia in vivo, which suggested that the miR-451 expressed at high levels in RBCs is independent of parasite infection. The weaker expression of miR-451 in higher parasitaemia, as detected by Northern blotting, is due to dilution of parasite RNAs, because this difference was abolished after adjusting the total RNAs loaded based on the number of blood cells. In addition, an ASO against miR-451, which normally inhibits miRNA activity [28-30], had no observable effects on the growth of *P. falciparum*. Several potential explanations need to be addressed in future studies: (1) the ASO failed to diffuse into the RBCs, presumably due to their integration in the cell membrane of RBCs and the presence of nuclease in medium; (2) the accumulation of miR-451 to a very high level in RBCs abolished the inhibitory function of the ASO, even the ASOs that could enter the cells; (3) the lower affinity of the locked nucleic acid (LNA/DNA) miR-451 ASO might influence the formation of stable complexes with the blocking oligonucleotide [31]. Further investigations are required to delineate the relationship between *P. falciparum *infection and human miRNA accumulation in RBCs.

## Abbreviations

siRNAs: short interfering RNAs; RISC: RNA-induced silencing complex; iRBCs: infected red blood cells; ASO: antisense oligonucleotide; WBC: white blood cells; has: Homo sapiens;LNA: locked nucleic acids

## Authors' contributions

XX carried out the construction and analysis of small RNA library, participated in the analysis of miR-451 expression and drafted the manuscript. QZ carried out Northern blot of miR-451 expression. LF was involved in the identification of small RNA. YH was responsible for the culture of *P. falciparum*. WP made contribution to the conception and design, revised the manuscript and finally approved the version of the manuscript for publication. All authors read and approved the final manuscript.
